# EARLY WARNING SIGNS AND CRISES IN ADRD/AD: VIEWS FROM LATINO/AFRICAN AMERICAN CAREGIVERS AND PROVIDERS

**DOI:** 10.1093/geroni/igad104.0815

**Published:** 2023-12-21

**Authors:** Melinda Kavanaugh

**Affiliations:** University of Wisconsin - Milwaukee, Milwaukee, Wisconsin, United States

## Abstract

Incidence of Alzheimer’s disease and related dementias (ADRD) in Latino and African American populations is significantly greater than for non-Hispanic Whites. Latino and African American caregiver families rely on family for care, including children and youth, and access formal care systems less frequently and at later stages compared to non-Latino White families. Yet we know little about what precipitates the eventual move to formal care. This study sought to understand whether African American and Latino families living with ADRD were able to identify Early Warning Signs (EWS) and potential crises in persons living with ADRD and explore differences in how families and health care professionals perceive EWS and crises. Semi-structured interviews were conducted with health care professionals (n = 26), Latino and African American adult (n = 36), and youth caregivers aged 10 – 19 (n = 31). Participants listed eight EWS (cooking, getting lost, forgetfulness, emotional/anger, repeating, bringing up the past) and defined Crisis as an event or series of events that led to seeking formal care, included getting lost, bad falls, and death of a loved one. Adults felt frustration, even at the beginning of EWS while youth found humor in the care. There were differences in stories shared within the same caregiver family; youth recognized EWS but reported that adults would not talk about it. Health care professionals identified EWS but believed families would be unable to identify EWS. This potential misperception needs further attention to reduce communication barriers around the care planning for this population.

